# Effects of *NR1H3* Genetic Variation on the Expression of Liver X Receptor α and the Progression of Alzheimer's Disease

**DOI:** 10.1371/journal.pone.0080700

**Published:** 2013-11-20

**Authors:** Teemu Natunen, Henna Martiskainen, Timo Sarajärvi, Seppo Helisalmi, Juha-Pekka Pursiheimo, Jayashree Viswanathan, Marjo Laitinen, Petra Mäkinen, Tarja Kauppinen, Tuomas Rauramaa, Ville Leinonen, Irina Alafuzoff, Annakaisa Haapasalo, Hilkka Soininen, Mikko Hiltunen

**Affiliations:** 1 Institute of Clinical Medicine – Neurology, University of Eastern Finland and Department of Neurology, Kuopio University Hospital, Kuopio, Finland; 2 Turku Centre for Biotechnology, University of Turku, Turku, Finland; 3 Department of Pathology, Kuopio University Hospital, Finland and Institute of Clinical Medicine, Unit of Pathology, University of Eastern Finland, Kuopio, Finland; 4 Institute of Clinical Medicine – Neurosurgery, University of Eastern Finland and Neurosurgery of NeuroCenter, Kuopio University Hospital, Kuopio, Finland; 5 Department of Immunology, Genetics and Pathology, Uppsala University, Uppsala, Sweden; Pasteur Institute of Lille, France

## Abstract

Alzheimer's disease (AD) has been postulated to involve defects in the clearance of amyloid-β (Aβ). Activation of liver X receptor α (LXRα) increases the expression of apolipoprotein E (ApoE) as well as cholesterol transporters ABCA1 and ABCG1, leading to augmented clearance of Aβ. We have previously shown that the C allele of rs7120118 in the *NR1H3* gene encoding LXRα reduces the risk of AD. Here, we wanted to assess whether the rs7120118 variation affects the progression of AD and modulates the expression of *NR1H3* and its downstream targets *APOE, ABCA1* and *ABCG1*.We utilized tissue samples from the inferior temporal cortex of 87 subjects, which were subdivided according to Braak staging into mild, moderate and severe AD groups on the basis of AD-related neurofibrillary pathology. *APOE* ε4 allele increased soluble Aβ42 levels in the tissue samples in a dose-dependent manner, but did not affect the expression status of *APOE*. In contrast, the CC genotype of rs7120118 was underrepresented in the severe group, although this result did not reach statistical significance. Also, patients with the CC genotype of rs7120118 showed significantly decreased soluble Aβ42 levels as compared to the patients with TT genotype. Although the severity of AD did not affect *NR1H3* expression, the mRNA levels of *NR1H3* among the patients with CT genotype of rs7120118 were significantly increased as compared to the patients with TT genotype. These results suggest that genetic variation in *NR1H3* modulates the expression of LXRα and the levels of soluble Aβ42.

## Introduction

Alzheimer's disease (AD), the most common cause of dementia in elderly, is a progressive neurodegenerative disease leading to severe memory impairment and finally to death. The major neuropathological hallmarks are the extracellular amyloid plaques and intracellular neurofibrillary tangles (NFTs) [1]. Aggregation of amyloid-β (Aβ) peptide to Aβ oligomers and finally to amyloid plaques has been postulated to trigger downstream events in AD, such as hyperphosphorylation of tau leading to the formation of NFTs, synaptic dysfunction, and loss of neurons in specific brain areas. Aβ is processed from amyloid precursor protein (APP) after sequential cleavage by β- and γ-secretases [2]. It has been suggested that the elevation of Aβ levels in the sporadic AD is due to decreased clearance rather than increased production of Aβ [3]. Interestingly, the pathogenesis of the familial form of AD was recently shown to involve not only increased Aβ production but also slower Aβ clearance rate from the cerebrospinal fluid (CSF), implying that similar mechanisms may in fact underlie both forms of the disease [4]. Several enzymes and pathways are involved in Aβ degradation and clearance [5]. Furthermore, the strongest genetic risk factor in sporadic AD, allelic variation in *APOE* gene has been linked to the clearance of Aβ [6]. Apolipoprotein E (ApoE) is the major apolipoprotein in the central nervous system (CNS) and it is produced mainly by astrocytes but also by microglia [7]. ApoE mediates the lipid transport between different tissue and cell types [8]. There are three different isoforms of ApoE, ApoE2, -3 and 4, and these are encoded by *APOE* alleles ε2, ε3 and ε4, respectively. *APOE* ε4 allele increases the risk of AD and decreases the age of onset as compared to the most common allele ε3. In contrast, ε2 decreases the risk and delays the age of onset [9]. A recent study using CSF biomarkers and PiB PET imaging showed that Aβ accumulation in the human brain corresponded to the *APOE* genotype in an isoform-dependent manner (ε4>ε3>ε2) [10]. Furthermore, the same study showed that ApoE4 is less efficient in Aβ clearance than ApoE3 in a mouse model expressing human ApoE isoforms [10].

Liver X receptor α (LXRα) is a ligand-activated transcription factor, which controls the expression of *APOE*, ATP-binding cassette transporter A1 (*ABCA1*) and G1 (*ABCG1*) and other genes involved lipid homeostasis [11]. LXRα is expressed particularly in the liver, but also in the brain. LXRα forms a heterodimer with retinoid X receptor (RXR) and agonists of both LXRα and RXR have been shown to increase ApoE-dependent Aβ clearance in AD mouse models [6], [12]. Cholesterol transporters, like ABCA1, increase lipidation of ApoE and this is crucial for the Aβ clearance induced by an LXRα agonist [13]. Recently, the RXR agonist bexarotene was shown to reduce soluble Aβ levels and amyloid plaque burden as well as to reverse the cognitive deficits in two different AD mouse models after short- and long-term administration of the drug [12]. In spite of the fact that other studies have partially failed to replicate these results [14]–[17], these data emphasize the central role of LXRα and RXR in the regulation of Aβ accumulation in AD pathogenesis.

We have previously studied the genetic alterations in the *NR1H3* gene encoding LXRα in Finnish AD case-control cohort [18]. We found that the single nucleotide polymorphism rs7120118, located in the intron between exons 6 and 7, showed a protective effect for the C allele carriers (OR = 0.70, 95% CI 0.53–0.93). Moreover, the total-tau and the phospho-tau levels in the CSF were decreased in AD patients carrying the C allele of rs7120118 [18]. Prompted by these results, we wanted to investigate the effects of rs7120118 polymorphism in *NR1H3* gene in a clinically and neuropathologically well-characterized brain sample set consisting of 87 subjects with different degree AD pathology. Since LXRα is considered as a potential drug target in AD [6], [12], it is important to investigate whether genetic alteration in *NR1H3*affects the expression of LXRα or its downstream targets. Here, we have determined the effects of the rs7120118 polymorphism on the expression of *NR1H3, APOE, ABCA1*, and *ABCG1* as well as the levels of soluble Aβ42 and β-secretase activity in the inferior temporal cortex of AD patients at different stages of the disease.

## Materials and Methods

### Neuropathological sample cohort

Human post-mortem brain samples were obtained from Kuopio University Hospital. This set included inferior temporal lobe samples from 87 older individuals investigated within memory clinic research projects and later autopsied and evaluated for AD pathology (21 males and 66 females; mean age 81 ± SD 8.6 years) (Table 1). The set was subdivided in three severity groups; mild (n = 46), moderate (n = 14) and severe (n = 27) according to Braak staging (0–2 = mild, 3–4 = moderate, 5–6 = severe) [19]. The subjects with Braak stage 0 are included to the mild group. Written informed consent from the next of kin was obtained to use brain samples for research purposes. The Ethics Committee of the Kuopio University Hospital and University of Eastern Finland has approved the study.

**Table 1:** Demographics and pathology of the cases.

Severity^a^		Braak stage	Number of cases/stage	Gender: males/females	Age at death, mean (years)	PMD^b^, mean (hours)	Brain weight, mean (g)
**MILD**	n = 46	0	8	17/29	80.7	17.7	1215
		1	17				
		2	21				
**MODERATE**	n = 14	3	7	2/12	83.5	15.9	1100
		4	7				
**SEVERE**	n = 27	5	16	2/25	81.6	6.5	1030
		6	11				

a) Classification to mild, moderate and severe groups according to Braak staging; 0–2 = mild, 3–4 = moderate, 5–6 = severe.

b) Post-mortem delay.

### CSF analysis of Aβ42, phosphorylated tau and total tau

The CSF levels of Aβ42 (n = 28), total tau (tot-tau, n = 27) and phosphorylated tau (p-tau, phosphorylated at Thr181, n = 27) of the AD patients were measured by using commercially available enzyme-linked immunosorbent assays (INNOTEST® β-AMYLOID_(1-42)_, tot-tau, INNOTEST hTAU Ag, p-tau, INNOTEST PHOSPHO-TAU_(181P)_, Innogenetics, Ghent, Belgium).

### DNA extraction from the frozen brain tissue samples

Frozen brain tissue samples were dissected from the inferior temporal lobe and homogenized to TRI-reagent (10 ml TRI-reagent/1 g brain tissue). DNA was isolated from 1 ml of Trizol homogenate. For the initial tissue homogenization, 0.5 ml of Back extraction buffer (4 M guanidine thiocyanate; 50 mM sodium citrate; 1 M Tris, pH 8.0) was used per 1.0 ml of TRI-reagent used. Back extraction buffer was added directly to the organic phase and samples were mixed by inversion for 15 sec and incubated for 10 min at the room temperature. Phase separation was performed by centrifugation at 12000× g for 15 min at +4°C. The upper aqueous phase containing DNA was transferred to a clean tube and the interphase and organic phases were saved at +4°C for subsequent protein extraction. DNA from the aqueous phase was precipitated by adding 0.5 ml of isopropanol per 1.0 ml of TRI Reagent used for the initial homogenization (1∶1). Samples were mixed by inversion and incubated for 30 min at −20°C. DNA was collected by centrifugation at 12000× g for 25 min at +4C° and the supernatant was removed. DNA pellets were washed twice with 1.0 ml of 75% ethanol and air-dried for 5 min. DNA samples were dissolved in 50 µl of Tris-EDTA buffer (10 mM Tris; 0.1 mM EDTA, pH 8.0).

### Genotyping of DNA samples

DNA samples extracted from the post-mortem brain tissue samples were genotyped for SNP rs7180118 in *NR1H3* gene using cycle sequencing. PCR was conducted around the rs7180118 site by using the following primers: 5′-GCTCTCCCCTCCTTCAGAAT-3′ and 5′-CACGGAATGAACACCTCAAA-3′. After purification, the PCR products were subsequently sequenced using BigDye™ Terminator sequencing kit (Applied Biosystems) and ABI PRISM® 3100 Genetic Analyzer (Applied Biosystems).

### Extraction and analysis of RNA from the frozen brain tissue samples

The first set including 24 brain samples was homogenized in TRI-reagent. Samples were treated twice with Tissuelyser (Qiagen) for 2 min at +4°C. Chloroform was added (one fifth of the volume of TRI-reagent) and the tubes were shaken vigorously. Mixtures were kept at room temperature for 5 min and the phase separation was performed by centrifugation at 12000× g for 20 min at +4°C. The aqueous phase was transferred to clean tubes and RNA was precipitated with 2-propanol by mixing and incubating at room temperature for 30 min. Samples were centrifuged at 12000× g for 25 min at +4°C and the pellets were washed twice with 75% ethanol. RNA pellets were air-dried and dissolved in RNAse-free H_2_O. RNA from the second set including 63 brain samples was extracted using Qiagen RNeasy Lipid Tissue Mini Kit (Qiagen). The quality of RNA samples was elucidated by determining the RNA integrity number (RIN) values using 2100 Bioanalyzer (Agilent). Subsequently, RNA samples were categorized into three subgroups according the RIN values; <4, <6 or <8. The distribution of RNA samples based on the RIN values among the whole sample set was 41.0% (RIN<4), 28.2% (RIN<6) and 30.8% (RIN<8). The distribution of RIN values in the TRI-reagent extracted samples was 13.0% (RIN<4), 47.8% (RIN<6) and 39.2% (RIN<8), while it was 50.0% (RIN<4), 24.1% (RIN<6) and 25.9% (RIN<8) in the RNeasy Lipid Tissue Mini Kit extracted samples. The post-mortem delay did not show correlation with the RIN values (Pearson's correlation, r = −0.09, p = 0.40). Equal amounts of total RNA samples were subjected to cDNA synthesis using Dynamo qPCR kit (Finnzymes). Subsequently, KAPA PROBE FAST qPCR kit using PCR primers and probes that were designed using Roche Universal Probe Library (www.roche-applied-science.com/sis/rtpcr/upl/ezhome.html) were used for the amplification of cDNA samples by real-time quantitative PCR (7900HT, Applied Biosystems). The primers and probes were the following: For NR1H3 GTTATAACCGGGAAGACTTTGC and AAACTCGGCATCATTGAGTTG, and probe: 80; for ABCA1 GGCCTACCAAGGGAGAAACT and TGTCATCACATGTCACATCCA, and probe:84; for ABCG1 TCAGGGACCTTTCCTATTCG and TTCCTTTCAGGAGGGTCTTGT, and probe:22; for ApoE GGACGAGGTGAAGGAGCA and CTGCAGGCGTATCTGCTG, and probe: 64; for GAPDH GCATCCTGGGCTACACTGA and CCAGCGTCAAAGGTGGAG, and probe:82; and for β-Actin CCAACCGCGAGAAGATGA and CCAGAGGCGTACAGGGATAG, and probe:11. Comparative ΔΔCt method was used to analyze the GAPDH-normalized *NR1H3, ABCA1, ABCG1* and *APOE* mRNA levels.

### β-secretase activity assay

Tissue samples from the temporal cortex were dissected from the post-mortem brain of AD patients. Frozen samples were mechanically homogenized in an ice bath in 400 µl of buffer B (20 mM Hepes pH 7.5, 150 mM KCl, 2 mM EGTA) containing 1:100 EDTA-free protease inhibitor cocktail (Thermo Scientific) and Halt™ phosphatase inhibitor cocktail (Thermo Scientific). After one-hour centrifugation in 100000×*g* (50.4 Ti rotor; Beckman) at +4°C, the supernatant ( = soluble fraction) was collected and stored at −70°C for soluble Aβ x-42 measurements. The remaining pellet was washed with buffer B followed by centrifugation at 100000×*g* (30 min, +4°C). The supernatant was discarded and the pellet was solubilized in buffer B containing 1% CHAPSO (3-[(3-Cholamidopropyl)dimethylammonio]-2-hydroxy-1-propanesulfonate; Cat #220202, Calbiochem) by rotation at +4°C for 1 hour and centrifuged at 100000×*g* (1 hour) to collect the supernatant ( = membrane fraction) for the β-secretase activity assay. β-Secretase Activity Assay Kit (Cat #K360-100, BioVision, CA, USA) was used to measure β-secretase activity from the membrane fraction according to the manufacturer's instructions. Briefly, equal amounts (1 µg) of membrane protein fractions were incubated at +37°C for 1 hour with the β-secretase-specific fluorogenic substrate peptide conjugated to fluorescent reporter molecules EDANS and DABCYL. The β-secretase inhibitor provided in the assay kit was mixed with recombinant β-secretase and incubated for 1 hour. At the same time, another β-secretase inhibitor, GL189 (H-VENstatineVAEF-NH_2_; Product #565780-500MG, Calbiochem) was used to validate the specificity of the β-secretase activity assay. GL189 was added (end concentration 150 µM) to five additional, randomly selected AD brain membrane fraction samples and incubated for 1 hour. Subsequently, the emitted light (510 nm) was detected on a fluorescence microplate reader (Wallac) after EDANS excitation at 355 nm. Readings obtained from the substrate (without secretase) were subtracted from the readings of the samples before calculating the fold change in β-secretase activity. Both inhibitors decreased the β-secretase activity on average by 60%.

### Soluble Aβ42 measurements from the frozen tissue samples

Aβ x-42 levels were measured from the soluble fraction of homogenized inferior temporal lobe tissue samples (see sample preparation for the β-secretase activity assay) using Human/Rat *β* Amyloid 42 (High-Sensitive; 292-64501) ELISA Kit (Wako). After 30-minute incubation at the room temperature, the reaction was terminated and the absorbance was measured at 450 nm using an ELISA microplate reader (BioRad). Protein concentrations of the soluble fractions were measured using BCA protein assay (Pierce), and the Aβ concentrations were normalized to these total protein concentrations in each sample.

### Statistical analysis

All the statistical analyses were performed using SPSS/Win (version 17.0). One-way analysis of variance (ANOVA), followed by Fisher's least significant difference (LSD) post-hoc test was used in experiments with more than one variable. Haploview 4.2 program was used to determine the D'- and r^2^-values of SNP pairs (CEU population) within the single haplotype block at the 3′-end of *NR1H3* gene. The target gene option in the miRWalk database was used to assess the predicted miRNA binding sites in the *NR1H3* gene (http://www.umm.uni-heidelberg.de/apps/zmf/mirwalk/predictedmirnagene.html). Correlations were assessed using Pearson's two-tailed correlation analysis. All values are reported as means ± standard deviation (SD). The level of statistical significance was defined as p<0.05.

## Results

### Biochemical characterization of the temporal cortex tissue samples reveals augmented β-secretase activity and increased soluble Aβ42 levels with respect to disease severity

**Figure 1:**
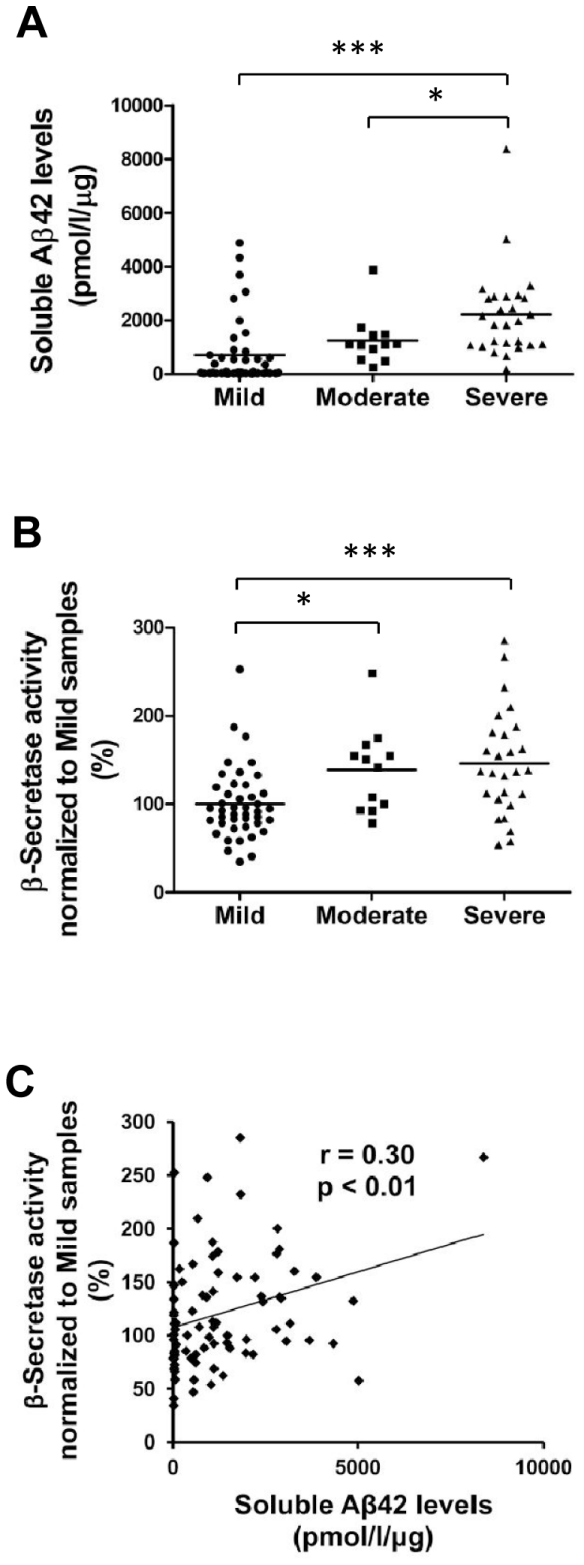
Soluble Aβ42 levels and β-secretase activity increase with respect to disease severity in the temporal cortex of AD brain. (A) Aβ42-ELISA measurements reveal a significant increase in the soluble Aβ42 levels in the severe group of temporal cortex samples as compared to the mild and moderate groups (***p<0.001, *p<0.05, ANOVA, LSD, mild n = 43; moderate n = 12; severe n = 27). (B) β-secretase activity assay indicates a significant increase in β-secretase activity in both moderate and severe groups as compared to the mild group of temporal cortex samples (***p<0.001, *p<0.05, ANOVA, LSD, mild n = 43; moderate n = 12; severe n = 27). (C) Soluble Aβ42 levels and β-secretase activity in the temporal cortex samples show significant correlation (p<0.01, Pearson two-tailed correlation, n = 85).

**Figure 2:**
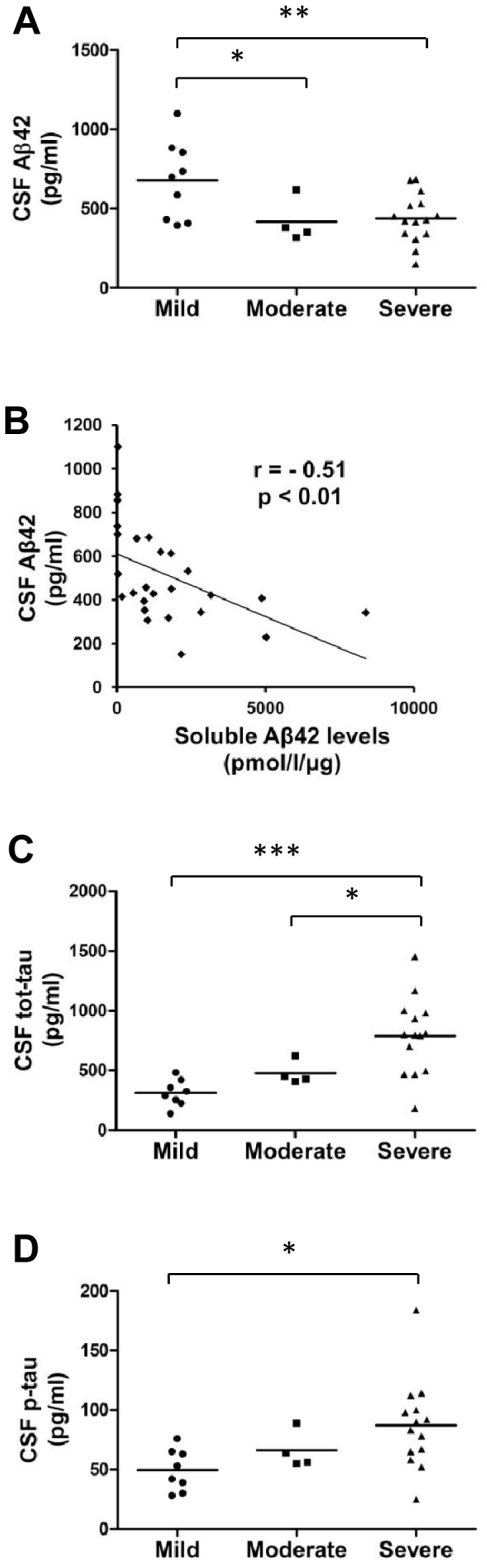
Aβ42 levels in the CSF decrease while tot-tau and p-tau levels increase with respect to disease progression in AD temporal cortex. (A) CSF Aβ42 levels are significantly decreased in both moderate and severe groups as compared to the mild group (**p<0.01, *p<0.05, ANOVA, LSD, mild n = 9; moderate n = 4; severe n = 15). (B) CSF Aβ42 levels and soluble Aβ42 levels indicate a significant negative correlation in the temporal cortex samples (p<0.01, Pearson two-tailed correlation, n = 26). (C) CSF tot-tau levels are significantly increased in the severe group as compared to the mild and moderate groups (***p<0.001, *p<0.05, ANOVA, LSD, mild n = 8; moderate n = 4; severe n = 14). (D) The p-tau levels in the CSF are significantly increased in the severe group as compared to the mild group (*p<0.05, ANOVA, LSD, mild n = 8; moderate n = 4; severe n = 14). Mean values are indicated in the graphs and each dot represents one individual.

Before determining the genetic effects of *APOE* and *NR1H3* gene variations in the brain, we first performed biochemical assessment of the brain sample set consisting of inferior temporal cortex samples from 87 subjects with neuropathologically well-defined AD neurofibrillary changes (Table 1). For the subsequent analyses, the sample set was subdivided according to Braak staging into three severity groups: mild (stages = 0–2), moderate (stages = 3 and 4), and severe AD (stages = 5 and 6) [19]. Soluble Aβ42 levels in the inferior temporal cortex samples in the severe group of AD patients were significantly increased as compared to mild (p = 0.00002) and moderate groups (p = 0.04) (Figure 1A). Moreover, β-secretase activity was significantly increased in both severe (p = 0.0002) and moderate (p = 0.02) groups as compared to the mild group (Figure 1B). Additionally, a significant positive correlation between soluble Aβ42 levels and β-secretase activity was detected (r = 0.30, p = 0.005) (Figure 1C). Aβ42, total-tau (tot-tau) and phospho-tau (p-tau) levels in the CSF were available for the subset of subjects. As expected, Aβ42 levels in the CSF were decreased in both moderate (p = 0.03) and severe (p = 0.006) groups as compared to the mild group (Figure 2A). A significant negative correlation between CSF Aβ42 levels and soluble Aβ42 levels in temporal cortex samples was observed (r = −0.51, p = 0.008) (Figure 2B). Furthermore, tot-tau levels in the CSF were increased in the severe group as compared to both moderate (p = 0.04) and mild groups (p = 0.0003) (Figure 2C). Also, p-tau levels in the CSF were significantly increased in the severe group (p = 0.01) as compared to the mild group (Figure 2D). Overall, these biochemical measurements in relation to the neurofibrillary changes indicating disease progression and severity reinforce the validity of this brain sample cohort for determining the effects of *APOE* and *NR1H3* gene variations on AD-related molecular events at different stages of AD.

### *APOE* ε4 allele increases the soluble Aβ42 levels in a dose-dependent manner in the AD brain tissue, but does not affect the expression status of *APOE*

**Figure 3:**
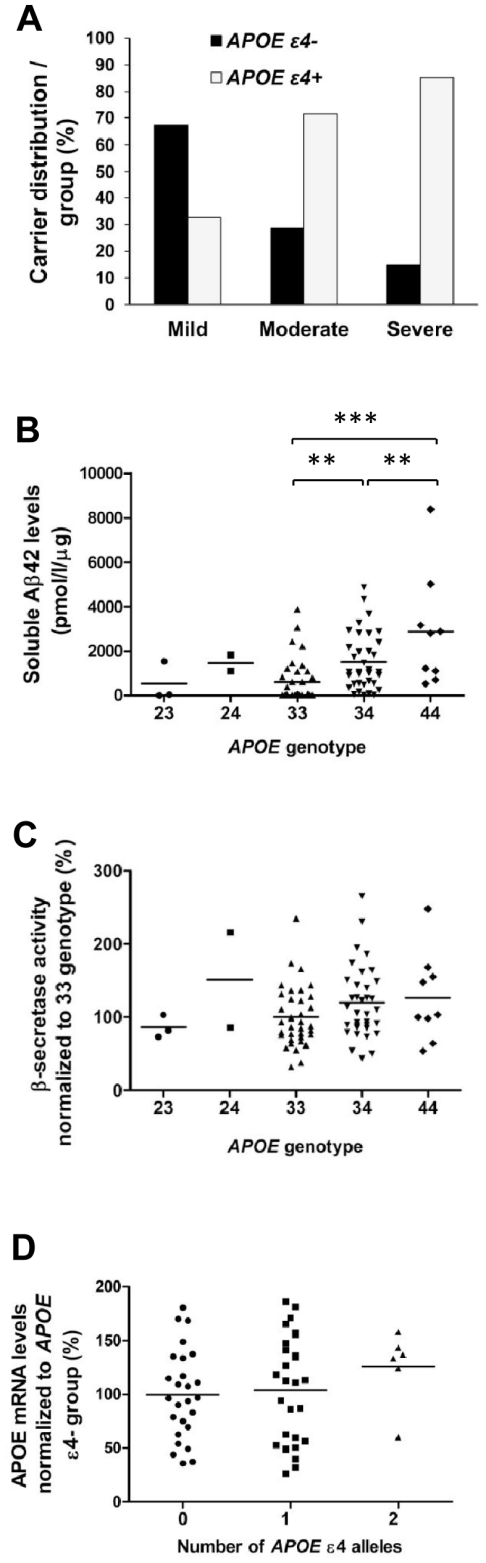
*APOE ε4* allele accelerates disease progression and increases soluble Aβ42 levels in the temporal cortex in a dose-dependent manner. (A) *APOE ε4* carriers are overrepresented in the moderate and severe groups (p<0.001, Pearson χ^2^, n =  (*APOE ε4-*/*ε4*+) mild 31/15; moderate 4/10; severe 4/23). (B) Aβ42-ELISA reveals a gene dose-dependent increase in the soluble Aβ42 levels in the temporal cortex of patients carrying the *APOE ε4* allele (***p<0.001, **p<0.01, ANOVA LSD, n =  3/2/36/35/9, *APOE* genotypes 23/24/33/34/44, respectively). (C) β-secretase activity assay shows a trend towards an increased β-secretase activity in relation to the copy number of *APOE ε4* allele (n =  3/2/36/35/9, *APOE* genotypes 23/24/33/34/44, respectively). (D) The qPCR analysis does not indicate significant changes in *APOE* mRNA levels in the temporal cortex with respect to the number of *APOE ε4* alleles (n = 26/28/6, number of *APOE* ε4 alleles 0/1/2, respectively). Mean values are indicated in the graphs and each dot represents one individual.

The frequency of *APOE* ε2/3/4 alleles in the AD brain sample set was 0.03/0.64/0.33, respectively, which is in line with our previous study [18]. The *APOE* ε4 allele carriers were overrepresented in moderate and severe groups as compared to the mild group (p = 0.00003) (Figure 3A). *APOE* ε4 allele increased soluble Aβ42 levels in a dose-dependent manner. This was indicated by the finding that the soluble Aβ42 levels were significantly increased in the AD patients with both *APOE* ε34 (p = 0.01) and ε44 genotypes (p = 0.00007) as compared to the ε33 genotype (Figure 3B). *APOE* ε4 allele also increased β-secretase activity in a dose-dependent manner, but this increase was not statistically significant (Figure 3C). qPCR analysis of *APOE* mRNA levels showed no significant changes with respect to the number of *APOE* ε4 alleles (Figure 3D). Altogether, these findings indicate that the *APOE* ε4 allele increases the soluble Aβ42 levels in the brain tissue in a dose-dependent manner, but does not affect the expression status of *APOE*.

### The CC genotype of rs7120118 in *NR1H3* gene associates with decreased soluble Aβ42 levels

**Figure 4:**
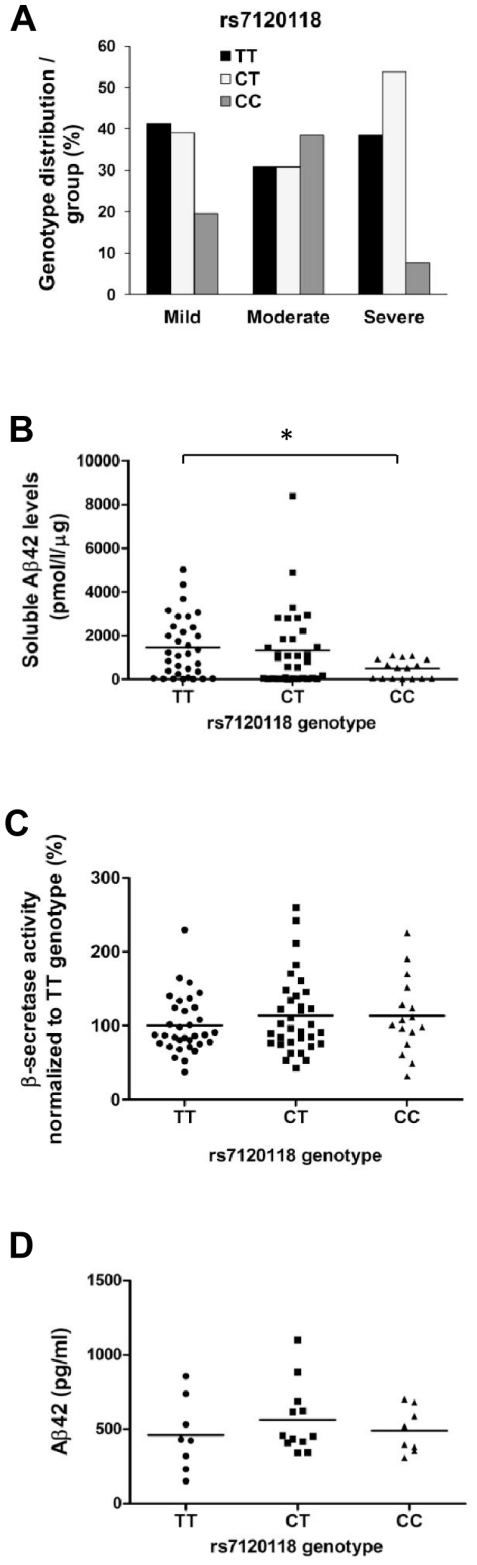
CC genotype of rs7120118 variation associates with decreased soluble Aβ42 levels in the temporal cortex. (A) The distribution of rs7120118 genotypes in subgroups divided by the severity of the disease (mild n = 19/18/9; moderate n = 4/4/5; severe n = 10/12/2, rs7120118 genotypes TT/CT/CC, respectively). (B) Aβ42-ELISA demonstrates a significant decrease in the soluble Aβ42 levels in the temporal cortex of patients with CC genotype as compared to the TT genotype (*p<0.05, ANOVA LSD, n = 33/34/16, rs7120118 genotypes TT/CT/CC, respectively). (C) *NR1H3* genotype does not affect β-secretase activity in the temporal cortex (n = 33/34/16, rs7120118 genotypes TT/CT/CC, respectively). (D) Aβ42 levels in the CSF are not significantly changed with respect to the rs7120118 genotype (n = 8/12/8, rs7120118 genotypes TT/CT/CC, respectively). Mean values are indicated in the graphs.

Next, we studied the effects of rs7120118 variation in the *NR1H3* gene on the progression of AD. The frequency of rs7120118 T/C alleles and TT/CT/CC genotypes was 0.59/0.41 and 0.38/0.42/0.20, respectively. This is in line with our previous data obtained from the Finnish AD patients in a clinic-based case-control cohort [18]. CC genotype was underrepresented in the severe group (Figure 4A), although this result did not reach statistical significance. Furthermore, soluble Aβ42 levels were significantly decreased among the patients with the CC genotype of rs7120118 (p = 0.03) as compared to the TT genotype (Figure 4B). However, β-secretase activity was not altered with respect to the rs7120118 genotypes (Figure 4C). Also, analysis of Aβ42 levels in the CSF revealed no significant changes with respect to the rs7120118 genotypes (Figure 4D). However, there was a trend towards a decrease in CSF tot-tau and p-tau levels among patients with rs7120118 CC genotype as compared to CT and TT genotypes (data not shown). These findings suggest that the CC genotype of rs7120118 might decrease the progression of AD and that the CC genotype associates with reduced soluble Aβ42 levels in the inferior temporal cortex.

### The CT genotype of rs7120118 associates with increased mRNA levels of *NR1H3*

**Figure 5:**
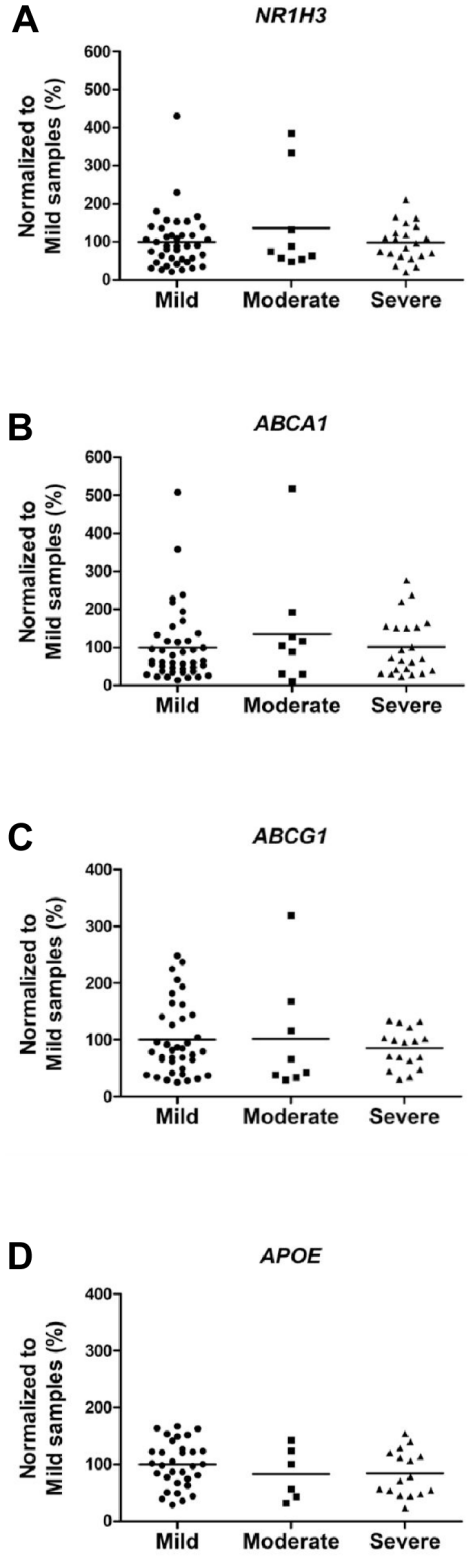
Severity of AD does not affect the mRNA levels of *NR1H3*, *ABCA1*, *ABCG1* or *APOE*. qPCR analysis of the mRNA levels of (A) *NR1H3*, (B) *ABCA1*, (C) *ABCG1* and (D) *APOE* mRNA levels does not reveal significant changes with respect to the severity of AD (*NR1H3* n = 40/9/21; *ABCA1* n = 42/9/22; *ABCG1* n = 38/8/17; *APOE* n = 32/6/16, mild/moderate/severe, respectively). Mean values are indicated in the graphs.

**Figure 6:**
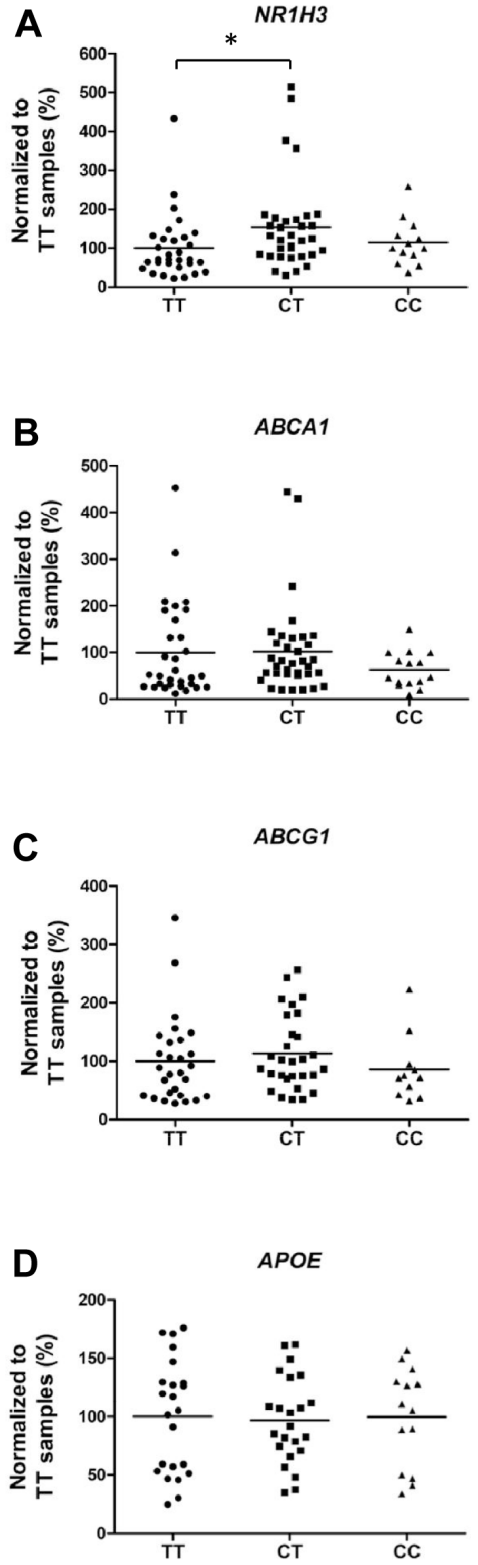
CT genotype of the rs7120118 variation increases the mRNA levels of *NR1H3* as compared to TT genotype. (A) qPCR analysis indicates ∼50% increase in the mRNA levels of *NR1H3* in the temporal cortex of AD patients with rs7120118 CT genotype as compared to TT genotype (*p<0.05, ANOVA LSD, n = 31/33/13, rs7120118 genotypes TT/CT/CC, respectively). (B–D) The mRNA levels of *ABCA1*, *ABCG1* and *APOE* do not change with respect to the rs7120118 genotype (*ABCA1* n = 31/35/15; *ABCG1* n = 28/29/11; *APOE* n = 23/23/14, rs7120118 genotypes TT/CT/CC, respectively). Mean values are indicated in the graphs.

Finally, we assessed whether the disease severity affects the expression of *NR1H3, ABCA1, ABCG1* and *APOE* in the brain tissue. qPCR analysis did not reveal significant effects on the mRNA levels of these four genes with respect to disease severity (Figure 5A–D). Consequently, we addressed the question whether rs7120118 variation affects the expression of *NR1H3, ABCA1, ABCG1*, and *APOE*. qPCR analysis showed a significant increase in the mRNA levels of *NR1H3* among AD patients with the CT genotype of rs7120118 (p = 0.02) as compared to TT genotype (Figure 6A). RNA quality (RIN values) did not significantly correlate with the altered *NR1H3* expression (Pearson's correlation, r = −0.20, p = 0.08). There were no significant alterations in the mRNA levels of *ABCA1*, *ABCG1* or *APOE* with respect to rs7120118 polymorphism (Figure 6B–D). In addition, a positive correlations between *NR1H3, ABCA1, ABCG1* and *APOE* mRNA levels was detected (*NR1H3* and *ABCA1*, Pearson's correlation r = 0.75, p = 5.9×10^−16^; *NR1H3* and *ABCG1*, r = 0.66, p = 1.1×10^−9^; *NR1H3* and *APOE*, r = 0.38, p = 0.004; *ABCA1* and *ABCG1*, r = 0.64, p = 2.6×10^−9^; *ABCA1* and *APOE*, r = 0.68, p = 3.4×10^−9^; *ABCG1* and *APOE*, r = 0.31, p = 0.03). Overall, these data suggest that the CT genotype of rs7120118 associates with increased mRNA levels of *NR1H3*, but the disease severity does not affect *NR1H3* expression.

## Discussion

In the present study, we have investigated the effects of rs7120118 variation on the expression of *NR1H3, APOE, ABCA1* and *ABCG1*, the levels of soluble Aβ42, and β-secretase activity in a sample set containing inferior temporal cortex samples of neuropathologically examined subjects with AD pathology. These samples were subdivided according to AD-related neurofibrillary pathology into mild, moderate and severe AD groups [19]. Clinical AD cases showing the Braak stage 0 were included to the mild group. The SNP rs7120118 was chosen for the analysis based on our previous study showing a protective effect for the C allele carries of rs7120118 alongside with reduced p-tau and tot-tau levels in the CSF of AD patients [18]. Although the *NR1H3* gene region has not invoked attention in terms of genetic association in the recent genome-wide association studies [20]–[22], LXRα is currently considered a potential drug target in AD [6], [12]. Thus, it is important to study whether alterations in *NR1H3* affect its own expression or the expression of its well-established downstream targets (*APOE, ABCA1* and *ABCG1*) known to play a role in the degradation and clearance of the Aβ peptide.

Before assessing the effects of the rs7120118 variation on gene expression and AD-related pathological events, we conducted a biochemical characterization of the tissue samples extracted from the inferior temporal cortex of subjects with AD pathology. Consequently, soluble Aβ42 levels were found to be gradually increased with respect to the progression of AD (mild, moderate, and severe), which in turn is in agreement with the results of previous studies showing that soluble, non-fibrillar Aβ levels are elevated in AD brain samples as compared to healthy controls [23]–[25]. Also, β-secretase activity was found to be higher in both moderate and severe groups as compared to the mild group. This finding is supported by the previous reports showing an augmented β-secretase activity in AD brain as compared to healthy controls [26]–[30]. Furthermore, we found a statistically significant correlation between β-secretase activity and soluble Aβ42 levels. These data are in line with the notion that β-secretase is the rate-limiting enzyme in Aβ generation and that augmented β-secretase activity leads to increased amyloid pathology. These findings also support the idea that β-secretase inhibition might be a useful option in the treatment of AD patients if started early enough. Several studies have shown that the Aβ42 levels in the CSF are decreased, while CSF tot-tau and p-tau levels are elevated in AD patients as compared to controls [31]–[34]. Here, Aβ42, tot-tau and p-tau levels in the CSF were available only from a subset of AD patients. In spite of the relatively small sample size, the CSF Aβ42 levels were significantly decreased, while the tot-tau and p-tau levels showed a significant increase with respect to the progression of AD. Importantly, there was a significant negative correlation between Aβ42 levels in the CSF and soluble Aβ42 levels in the temporal cortex. Collectively, these findings validated the utilization of these biochemical outcome measures in the brain tissue samples in the subsequent determination of the effects of *APOE* and *NR1H3* gene variations.

*APOE* ε4 allele is the major genetic risk factor for AD, which decreases the age of onset in a gene dose-dependent manner and increases Aβ deposition in the brain [9]. In the present study, *APOE* ε4 carriers were overrepresented in the moderate and severe groups of AD patients. Moreover, the *APOE* ε4 allele increased the soluble Aβ42 levels in a dose-dependent manner. This was an anticipated outcome as the previous studies have shown that *APOE* ε4 allele increases Aβ deposition in the cerebral cortex of AD brain [35] as well as in cognitively normal aging brain [36]. It has been shown that *APOE* ε4 allele is associated with reduced levels of Aβ42 in the CSF [37], which is considered to reflect increased Aβ42 levels in the brain. Furthermore, a recent study showed that ApoE4 is less efficient in Aβ clearance than ApoE3 in a mouse model expressing human ApoE isoforms [10]. Previous studies have assessed the ApoE protein levels in post-mortem brain samples with contrasting results, but this may partly be due to the different brain regions used in the assessments [38]–[43]. Only a few studies have elucidated *APOE* mRNA levels in the post-mortem brain samples [42]–[44]. In the present study, *APOE* mRNA levels showed an increasing trend among the AD patients homozygous for *APOE* ε4 as compared to patients heterozygous for *APOE* ε4 or with no ε4 allele. This finding is in agreement with a previous study showing that *APOE* mRNA levels are increased in the temporal lobe of AD patients carrying the *APOE* ε4 allele [45]. Taken together, our data suggest that *APOE* ε4 allele increases the soluble Aβ42 levels in the brain tissue in a dose-dependent manner, but does not significantly affect the expression of *APOE.*

Previously, gender-, age-, and *APOE*-adjusted logistic regression analysis revealed a protective effect for the C allele carriers of rs7120118 among a Finnish clinic-based AD-control cohort, indicating that these patients have a decreased risk for AD [18]. Consistent with this finding, the CC genotype of rs7120118 was found to be underrepresented in the severe group in the present study. This result, however, did not reach statistical significance. Moreover, there were proportionally more subjects with the CC genotype in the moderate group as compared to the mild group, arguing against an additive effect for the C allele in this sample set. Nevertheless, the protective effect of the C allele of rs7120118 was supported by the biochemical measurements showing significantly decreased soluble Aβ42 levels in the brain of AD patients with rs7120118 CC genotype as compared to the TT genotype. β-secretase activity was not affected by the rs7120118 variation, suggesting that the decrease in the soluble levels of Aβ42 may be linked to increased clearance and/or degradation rather than decreased production of Aβ. This is a plausible assumption considering that the LXRα agonists increase the clearance of Aβ [6]. The Aβ42 levels in the CSF did not show significant alteration with respect to the rs7120118 genotypes, but this could be due to the small number of CSF samples available for the present analysis. Since the mRNA levels of *NR1H3, ABCA1, ABCG1*, and *APOE* did not change with respect to the severity of AD, we were able to address the question whether the rs7120118 variation affects the mRNA levels of these genes. Consequently, the mRNA levels of *NR1H3* were found to be significantly increased among the AD patients with the rs7120118 CT genotype as compared to the TT genotype. However, since the mRNA levels of *NR1H3* in the subjects with CC genotype were in a similar level as in the subjects with TT genotype, it is questionable whether the expressional increase of *NR1H3*observed in the CT genotype group is biologically relevant. In line with this, increased mRNA levels of *NR1H3* in the rs7120118 CT genotype group did not affect the expression of well-established *NR1H3* downstream targets, *ABCA1*, *ABCG1* or *APOE* when analyzed with respect to rs7120118 variation. However, a significant positive correlation between the mRNA levels of *NR1H3, ABCA1, ABCG1*, and *APOE* was observed, indicating that at an individual level, changes in the expression of *NR1H3* are subsequently reflected on the expression of its downstream targets. Finally, it is unclear how the observed *NR1H3* results from post-mortem samples reflect the situation in the living human brain. For example, it is possible that post-mortem-related degradation processes have affected the observed results. Although there was variation with respect to RNA quality, long post-mortem delays did not correlate with reduced RNA quality (RIN values). Also, variation in the RNA quality did not correlate with altered *NR1H3* expression.

LXRα is involved in the control of lipid homeostasis and inflammation, while the activation of LXRα-related downstream targets has beneficial effects in pathologic conditions, such as atherosclerosis [46], inflammation [47], and AD [48]. However, long-term activation of LXRα may lead to adverse side effects, including hepatic steatosis [49]. Thus, it is rational that there exist mechanisms, which can efficiently regulate the activation and/or expression of LXRα. A complex regulation system that was recently presented suggests that LXRα autoregulates its own expression via the induction of SREBP-1c, which in turn up-regulates miRNA hsa-miR-613[50]. This miRNA has a binding site at the 3′UTR of *NR1H3* and thus is able to repress the expression of LXRα. Since rs7120118 resides at a large haplotype block at the 3′UTR of the *NR1H3* gene, in which the SNPs are in strong linkage disequilibrium (D' = 0.92–1.0, r^2^ = 0.03–0.96), it is possible that genetic variation within or in the vicinity of miRNA binding site(s) could alter miRNA binding and thus affect the expression of LXRα. In fact, SNP rs375078947 locates immediately next to the 5′ end of the binding site of miRNA hsa-miR-613 and thus it is possible that this polymorphism could affect the binding affinity of this miRNA to *NR1H3*. Apart from the already validated binding of miRNA hsa-miR-613 to the *NR1H3* [50], a search on a database predicting the miRNA targets (miRWalk) revealed 25 putative miRNA binding sites in the 3′UTR of *NR1H3*, suggesting that miRNA-mediated regulation of LXRα expression could extend beyond hsa-miR-613.

Taken together, the results of the present study suggest that rs7120118 polymorphism in *NR1H3* affects LXRα expression and the soluble levels of Aβ42 in the temporal cortex of AD patients. Particularly, the CC genotype of the rs7120118 variation, which associated with decreased AD risk in our previous study, is linked to decreased soluble Aβ42 levels in AD brain. Although further studies are needed to unravel the underlying molecular mechanisms related to these findings, it is possible that they reflect changes in Aβ clearance mechanisms that, depending on the genetic variation, may either enhance or decelerate the disease pathogenesis.Thus, it is possible that AD patients with different *APOE* and *NR1H3* genotypes respond differently to LXRα agonist or other ApoE-related treatments. These notions emphasize the importance of determining the genetic profiles of these potential drug targets when designing treatment options for individual AD patients.
